# Peer Review of “Safety and Efficacy of Chimeric Antigen Receptor T-Cell Therapy for Recurrent Glioblastoma: An Augmented Meta-Analysis of Phase 1 Clinical Trials (Preprint)”

**DOI:** 10.2196/71293

**Published:** 2025-01-24

**Authors:** Vanessa Fairhurst, Randa Salah Gomaa Mahmoud, Toba Olatoye, Sylvester Sakilay

**Affiliations:** 1 PREreview, -, Portland, OR, United States, 1 123456789; 2 Faculty of Human Medicine, Zagazig University, Zagazig, Egypt; 3 University of Ilorin, Ilorin, Nigeria

**Keywords:** CAR T-cell therapy, cancer, glioblastoma, brain tumor, meta-analysis, chimeric antigen receptor

*This is a peer-review report for the preprint “Safety and Efficacy of Chimeric Antigen Receptor T-cell Therapy for Recurrent Glioblastoma: An Augmented Meta-Analysis of Phase 1 Clinical Trials*.”

This review is the result of a virtual collaborative live review discussion organized and hosted by PREreview and JMIR Publications on Dec 12, 2024. The discussion was joined by 11 people: 3 facilitators, 1 member of the JMIR Publications team, and 7 live review participants including 3 who agreed to be named but did not assist in compiling the final review: Eudora Nwanaforo, Kelechi Elechi, and Murtala Haruna Bawa. The authors of this review have dedicated additional asynchronous time over the course of 2 weeks to help compose this final report using the notes from the live review. We thank all participants who contributed to the discussion and made it possible for us to provide feedback on this preprint.

## Summary

The study [1] was designed to address the limitations of previous studies and evaluate the safety and efficacy of chimeric antigen receptor (CAR) T-cell therapy for recurrent glioblastoma. The results of this study are predictive rather than confirmatory. CAR T-cell therapy for glioblastoma was not predicted to significantly improve survival or achieve substantial complete responses. Stable disease rates were modest, while disease progression was notable. Adverse events, especially CAR T-cell therapy–related encephalopathy, raise safety concerns. Overall survival was 6.49 months in patients receiving CAR T-cell therapy after augmented analysis, and only 80% of patients exhibited this outcome. It was not statistically different from the median overall survival observed in patients with recurrent glioblastoma undergoing standard treatment, thereby indicating that CAR T-cell therapy, in its current form, does not offer substantially improved survival compared to standard treatments. Further trials and refinements are needed to enhance CAR T-cell therapy’s effectiveness and safety in glioblastoma treatment. An interesting fact is that a novel statistical technique (augmented meta-analyses) was used in this study. It was a combination of a cross-sectional (quantitative) and augmented meta-analysis (qualitative).

## List of Major Concerns and Feedback

### Methods

#### Augmented Meta-Analysis

- This section is limited in its description of the methodology used in the study. It would be helpful to include more information on the machine learning model or language model used to generate the extra cases.
- The title and aim specify that the study focuses on recurrent glioblastoma, but this specificity is not reflected in the inclusion criteria. It would be helpful to adjust the inclusion criteria to explicitly state that the study is targeting patients with recurrent glioblastoma. This will align the methodology with the aim as stated.
- The inclusion criteria do not specify that patients are in phase 1 clinical trials, where safety is a primary focus. Clearly state in the inclusion criteria that patients are part of phase 1 clinical trials. This will provide context for the study’s focus on safety.
- There is no reference to the earlier use of augmented meta-analysis in cancer or medical research, nor is it explicitly stated if this is a new application. If augmented meta-analysis has been previously applied, cite relevant references. If this is its first application, explicitly state so and highlight its novelty.

### Results

#### Literature Review and Risk of Bias Assessment Section

- It would be helpful to add the details of Figure 1 and Table 1 that explain the details of the cause of exclusion, the results of the Newcastle Ottawa Scale, which study reached the high-quality level, etc.

### Discussion

- It is important to add a comparison between the mean overall survival for patients with glioblastoma who underwent CAR T-cell therapy and the median overall survival observed in patients receiving the standard protocol for recurrent glioblastoma treatment to the Results section, as this comparison is mentioned in the first paragraph of the Discussion section.

### Reproducibility of the Study

- The data presented in the study are beneficial for reproducibility except for the augmented meta-analysis, which is hindered by the lack of clear documentation on the large language model settings.
- The details of the augmented meta-analysis are not available. Provide access to the source code or methodological details for augmented meta-analysis, either as supplementary material or a public repository link. Transparency will strengthen the study’s reproducibility.

## List of Minor Concerns and Feedback

### Concerns With Techniques/Analyses

- Abbreviations like “IL-13Ralpha-2,” “EGFRvIII,” “HER2,” and “HephA2” are not identified in the Included Study Characteristics section. Expand the abbreviations and provide their full names (eg, “Interleukin-13 Receptor Subunit Alpha-2”) when first mentioned. This ensures clarity for readers not familiar with the terms.
- The last line of the large language model statement on page 16 does not explain how augmented meta-analysis was applied. Elaborate on how augmented meta-analysis was applied, especially in terms of methodology and integration with the study data.

### Figures and Tables

- The screening section in Figure 1 is missing a rectangle to indicate the exclusion of 300 records. Update it using the PRISMA (Preferred Reporting Items for Systematic Reviews and Meta-Analyses) flowchart to include a rectangle that details the 300 excluded records and ensures the causes of exclusion are clearly stated.
- The reasons for exclusion are not detailed in the PRISMA flowchart. Follow PRISMA guidelines to specify the causes of exclusion, such as duplicates, irrelevance, or incomplete data, within the flowchart.
- Comments following Figure 1 are not in line with its instructions. Restructure the comments to follow the instructions and present the details of the research study accordingly.

### Additional Comments

- No reference is provided for the trim-and-fill method mentioned in the augmented meta-analysis of overall survival (page 10). Cite a relevant source, such as [2] or another appropriate reference.
- The Cochrane Handbook (Part 2, Chapter 9) should be referenced in the Statistical Analysis section and its numbered reference cited in the text.
- References in the third paragraph of the Introduction mix meta-analyses and clinical trials without clear distinction. Rearrange and clarify the references while ensuring that references to meta-analyses and clinical trials are grouped and contextualized appropriately to avoid confusion.
- Repetition of the sentence “Egger’s test for publication bias could not be performed since the number of included studies in this outcome was less than ten” could be avoided by mentioning it once in the Methods section as the total number of the included studies is 8.
- In addition, the repetition of the sentence “The wide range of the 95% confidence interval was suggestive of data sparsity, so augmented meta-analysis was indicated before making conclusions” could be avoided by mentioning it once in the Augmented Meta-Analysis section of the Methods.

## Abbreviations

CAR: chimeric antigen receptor
PRISMA: Preferred Reporting Items for Systematic Reviews and Meta-Analyses

## References

[1] Azzam AY, Morsy MM, Azab MA (2024). Safety and efficacy of chimeric antigen receptor T-cell therapy for recurrent glioblastoma: an augmented meta-analysis of phase 1 clinical trials. medRxiv.

[2] Shi L, Lin L (2019). The trim-and-fill method for publication bias: practical guidelines and recommendations based on a large database of meta-analyses. Medicine (Baltimore).

